# Neural processing of biological motion in autism: An investigation of brain activity and effective connectivity

**DOI:** 10.1038/s41598-017-05786-z

**Published:** 2017-07-17

**Authors:** Kaat Alaerts, Stephan P. Swinnen, Nicole Wenderoth

**Affiliations:** 1 Department of Rehabilitation Sciences, Group Biomedical Sciences, Neuromotor Rehabilitation Research Group, University of Leuven, KU Leuven, Belgium; 2 Department of Kinesiology, Group Biomedical Sciences, Movement Control and Neuroplasticity Research Group, University of Leuven, KU Leuven, Belgium; 3 Department of Health Sciences and Technology, Neural Control of Movement Lab, ETH Zurich, Switzerland

## Abstract

The superior temporal sulcus (STS) forms a key region for social information processing and disruptions of its function have been associated with socio-communicative impairments characteristic of autism spectrum disorders (ASD). Task-based fMRI was applied in 15 adults with ASD and 15 matched typical-controls (TC) to explore differences in activity and effective connectivity of STS while discriminating either ‘intact’ versus ‘scrambled’ biological motion point light displays (*explicit* processing) or responding to a color-change while the ‘intact’ versus ‘scrambled’ nature of the stimulus was irrelevant for the task (*implicit* processing). STS responded stronger to ‘intact’ than ‘scrambled’ stimuli in both groups, indicating that the basic encoding of ‘biological’ versus ‘non-biological’ motion seems to be intact in ASD. Only in the TC-group however, explicit attention to the biological motion content induced an augmentation of STS-activity, which was not observed in the ASD-group. Overall, these findings suggest an inadequacy to recruit STS upon task demand in ASD, rather than a generalized alteration in STS neural processing. The importance of attention orienting for recruiting relevant neural resources was further underlined by the observation that connectivity between STS and medial prefrontal cortex (mPFC), a key region in attention regulation, effectively modulated STS-recruitment in the ASD-group.

## Introduction

Humans have the remarkable ability to perceive and understand facial expressions, body language and intentions of others in a seemingly effortless way. Nowadays, research is increasingly focusing on exploring the link between one’s ability to perceive and interpret non-verbal cues originating from the communicator’s face and body and the development of social interaction skills. This is of particular relevance for patient populations with specific impairments in the social-communicative domain such as autism spectrum disorders (ASDs). While facial expressions form a salient source of input for conveying socially communicative information, other sources, such as the communicator’s body language or “bodily kinematics” - are equally important, especially when facial expressions are inconsistent or unavailable to the observer. In vision research, point-light displays (PLDs), representing biological motion solely by a set of small lights or markers attached to the major joints of an actor’s body, provide a widely adopted paradigm to investigate bodily motion perception^1^.

From a behavioral perspective, studies consistently showed that people with ASD display difficulties with extracting higher-order information such as emotional content or gestures from PLDs^2–8^. Also a variety of task paradigms have been adopted to explore more basic biological motion processing abilities in people with ASD. While some reported marked impairments, others found no specific deficiency in biological motion perception in ASD. For example, for tasks involving the labeling of low-level features such as the action of the presented PLD^2, 3, 5, 9^ or identifying biological motion from object motion^10^, a majority of studies found no specific deficit in ASD (but see ref. 4). Also on tasks involving the detection of biological motion embedded in noise, a majority of studies found no evidence of reduced performance in participants with ASD^11–13^ (but see ref. 14). Other basic biological motion tasks however, involving the identification of ‘biological’ from ‘scrambled’ dot motion consistently showed ASD-related impairments as revealed by reduced accuracy and/or reaction times for discriminating ‘intact’ from ‘scrambled’ PLD^6, 15–18^.

At the neural level, a number of studies consistently identified a significant involvement of the superior temporal sulcus region (STS) in biological motion processing from PLD^19–25^ (most prominently in right STS^21, 26, 27^). Interestingly, the STS is also known to form a key ‘hub’ in social information processing by connecting distinct social brain networks underlying theory of mind (amygdala-prefrontal network) and action/emotion understanding (action observation network or mirror neuron system)^28^. Particularly, together with the fusiform gyrus, orbitofrontal cortex, medial prefrontal cortex and amygdala, the STS is suggested to be involved in multiple aspects of social perception, including mentalizing, face processing, and social reward processes^29–32^. Further, the posterior STS is also known to provide the main visual input to the fronto-parietal regions of the action observation network (inferior frontal gyrus, inferior parietal lobule)^33^ which is suggested to provide a direct ‘motor matching’ mechanism for understanding other people’s actions and emotions, thereby subserving aspects of embodied cognition^34, 35^.

Considering the important role of the STS in distinct aspects of social cognition, a number of studies have investigated the contribution of STS in the neural expression of ASD. At the structural level, several studies have shown alterations in STS regions in ASD^36–41^ and, also at the functional level, evidence is accumulating^32, 42–45^. Among these, and related to the relative specialization of the STS in processing non-verbal cues, a handful of studies specifically explored the differential recruitment of STS during biological motion perception in ASD^7, 18, 46–49^. For example, in an initial fMRI study by Herrington *et al*.^47^, adult patients with Asperger syndrome were presented with ‘intact’ PLD walkers or scrambled versions of the same stimuli and were asked to indicate the movement direction of the walker. While behavioral performance was equal across groups, the Asperger group displayed significant reductions in neural activity in a widespread visual-temporal-parietal network, including middle and superior temporal gyri encompassing the STS. Freitag *et al*.^18^ used a similar task in which participants were instructed to assemble the presented intact or scrambled PLD to a figure for later report, and showed that patients with ASD displayed less activity in a similar network including right middle temporal gyrus, adjacent to the STS. Using a coherent motion paradigm, Koldewyn *et al*.^48^ also showed that patients with ASD displayed reduced activity in a network of regions, including the inferior parietal sulcus, right inferior frontal gyrus, anterior cingulate and right STS when contrasting blocks of coherent dot motion with blocks of PLD biological motion embedded in coherent dot motion. In a more recent study from our group^7^, patients with ASD and control subjects were asked to label emotional content from biological PLD and activity in a similar set of regions (bilateral STS, inferior parietal lobule and middle occipital gyrus) was significantly higher in the control group, compared to the ASD group.

Overall, these previous studies consistently showed that the recruitment of STS regions is diminished in ASD during *explicit* processing of the biological motion content in PLD stimuli. It remains unclear however, whether this pattern of results reflects an inadequacy of ASD patients to recruit the necessary neural resources (STS) upon task demand, or whether neural processing at the level of STS is aberrant irrespective of task instruction. This issue seems relevant in the context of the influential ‘social motivation theory’ of ASD^50^, postulating that patients with ASD may be primarily characterized by a reduced ability to *recruit* the relevant socio-cognitive skills to perform social tasks (due to impairments in social attention orienting), but that the underlying socio-cognitive resources may be relatively intact.

To systematically explore the relevance of task demand and attention orienting in biological motion processing in ASD, the present study adopted a 2 × 2 factorial design to assess differential modulations of STS activity during *explicit versus implicit* processing of *intact versus scrambled* PLD motion in patients with ASD and typical control (TC) participants. Also differential changes in effective connectivity of the right STS are explored. During the *explicit* biological motion condition, participants were instructed to explicitly orient attention to the ‘biological’ content embedded in the PLD stimuli by asking them to actively discriminate ‘intact’ from ‘scrambled’ PLD stimuli^6, 15–18^. During the *implicit* task condition on the other hand, the same PLD stimuli were presented, but no task instruction was given to explicitly attend to the biological motion content of the stimuli. Instead, during the implicit condition, participants were required to orient attention to random color changes in the moving point lights.

Overall, and consistent with previous studies^21, 26, 51^, the typical control (TC) group was expected to display higher STS activity for perceiving ‘intact’, compared to ‘scrambled’ PLD, irrespective of whether attention was explicitly oriented to the ‘biological’ content embedded in the PLD. Further, we additionally hypothesized that, in the TC group, STS activity would be significantly enhanced when participants are explicitly instructed to orient attention to the biological motion content in the PLD, compared to the implicit condition where attention is oriented away from the biological motion content. In relation to ASD, a primary aim was to explore whether differential STS activity for processing intact versus scrambled PLD is also present in the ASD group, and in particular, whether this pattern is modulated by task demand. If neural processing at the level of STS is overall aberrant, we expected STS recruitment to be overall reduced in the ASD group compared to the TC group, irrespective of stimulus- or task-demand. On the other hand, if ASD is primarily reflected by an inadequacy to recruit the necessary neural resources upon task demand, we expected STS recruitment to be specifically altered in the explicit attention orienting condition, not in the implicit condition.

## Results

### Behavioral Performance

**Figure 1:**
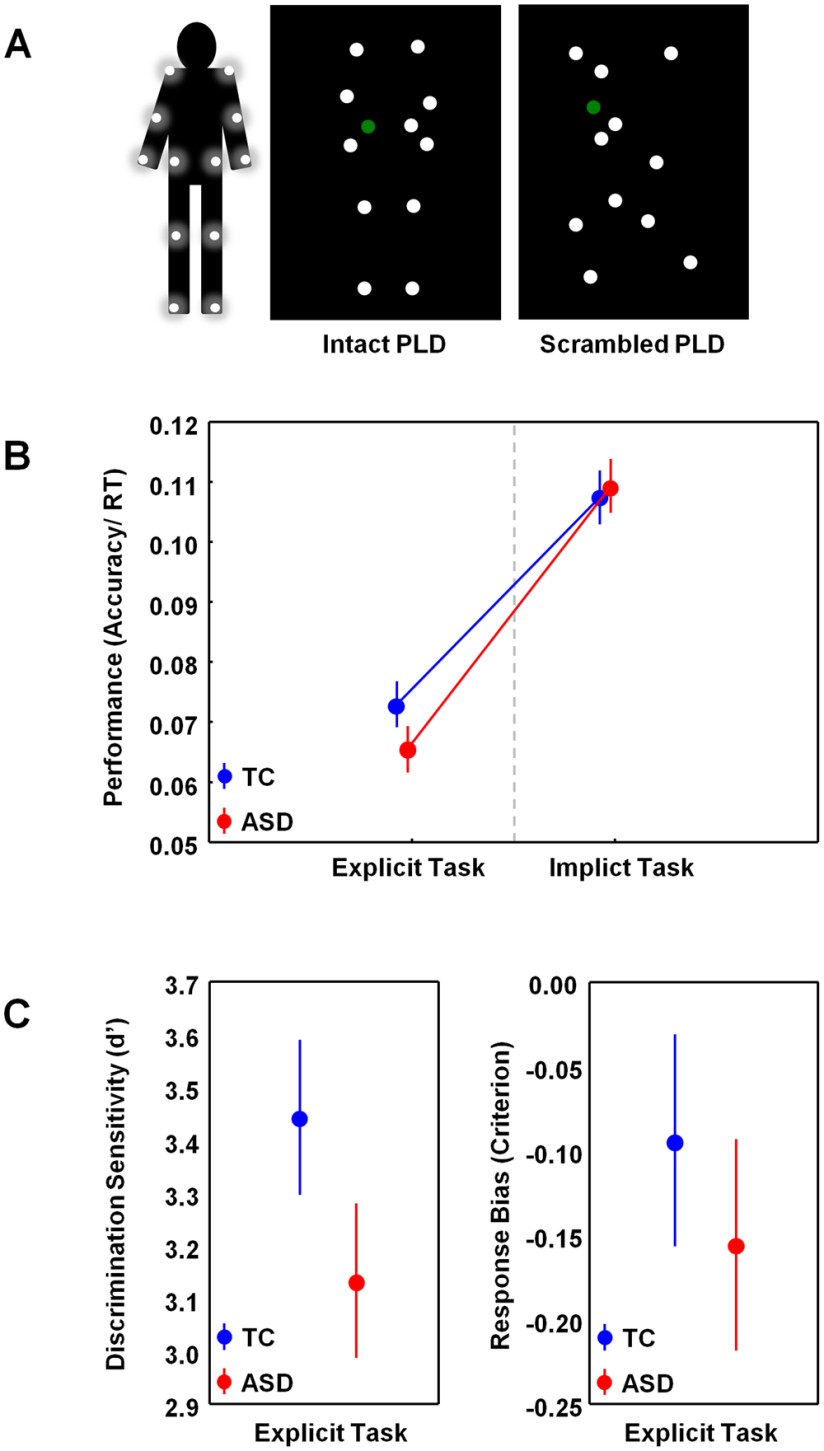
Experimental task. Panel A visualizes an example of an intact and scrambled point light display (PLD) stimulus. In the explicit task condition, participants were instructed to indicate as fast and accurate as possible whether the presented PLD represented ‘a person’ or ‘not a person’. In the implicit task condition, participants were instructed to indicate color changes in the moving dots (at a random time point, one dot briefly changed color to either ‘red’ or ‘green’). Panel B visualizes performance scores (accuracy/reaction time) for the explicit and implicit task condition, separately for each group (TC, ASD). Panel C visualizes discrimination sensitivity (d’) and response bias scores (criterion) for the explicit task condition, separately for each group (TC, ASD). Vertical bars denote +/− standard errors.

Behavioral performance was assessed on an explicit biological motion perception task involving the discrimination of intact from scrambled versions of biological point light display (PLD) stimuli (*explicit condition*) (Fig. 1A), as well as on an implicit task condition involving the indication of color changes in the moving point lights (*implicit condition*).

Performance indices (accuracy/RT) are displayed separately for each group (TC, ASD) and task condition (explicit, implicit) in Fig. 1B. A repeated-measures ANOVA analysis revealed a significant ‘group × task’ interaction (F(1,28) = 4.67; p < 0.05), indicating higher performance in the TC group, compared to the ASD group for detecting biological motion (explicit condition), but not for the implicit task condition, requiring the detection of color changes. Note that exclusion of the TC participant with a high self-reported SRS-score (see Methods and Supplementary Figure 1A) further enlarged the identified ‘group × task’ interaction effect (F(1,27) = 7.62; p = 0.01) (Supplementary Figure 1B).

For the explicit biological motion task, we also calculated (i) the discrimination sensitivity index (d’) (higher d’ indicates that ‘biological motion’ was more readily detected) and (ii) the response bias or criterion (criterion scores smaller than zero indicate a bias to respond ‘person’) (more detailed information on these measures is provided in the Methods). Discrimination sensitivity and response bias scores are displayed separately for each group (TC, ASD) in Fig. 1C.

Although discrimination sensitivity (d’) was tentatively higher in the TC group compared to the ASD group, the group difference failed to reach significance (with outlier TC participant: Z = 1.49; p = 0.13; Cohen’s d = 0.55) (without outlier TC participant: Z = 1.77; p = 0.07; Cohen’s d = 0.68). Also in terms of response bias (criterion), no significant group differences were observed, indicating no differential tendency to label the presented PLD as ‘biological’ (intact) or ‘scrambled’ (with outlier TC participant: t(28) = 0.70; p = 0.49; Cohen’s d = 0.25) (without outlier TC participant: t(27) = 1.27; p = 0.23; Cohen’s d = 0.47). Note however that only in the ASD group (t(14) = −2.48; p < 0.05), not in the TC group (t(14) = −1.49; p = 0.16), criterion scores were significantly smaller than zero (indicating a bias to respond ‘biological’).

### Whole-brain analysis of task-related activity during biological motion processing

**Figure 2:**
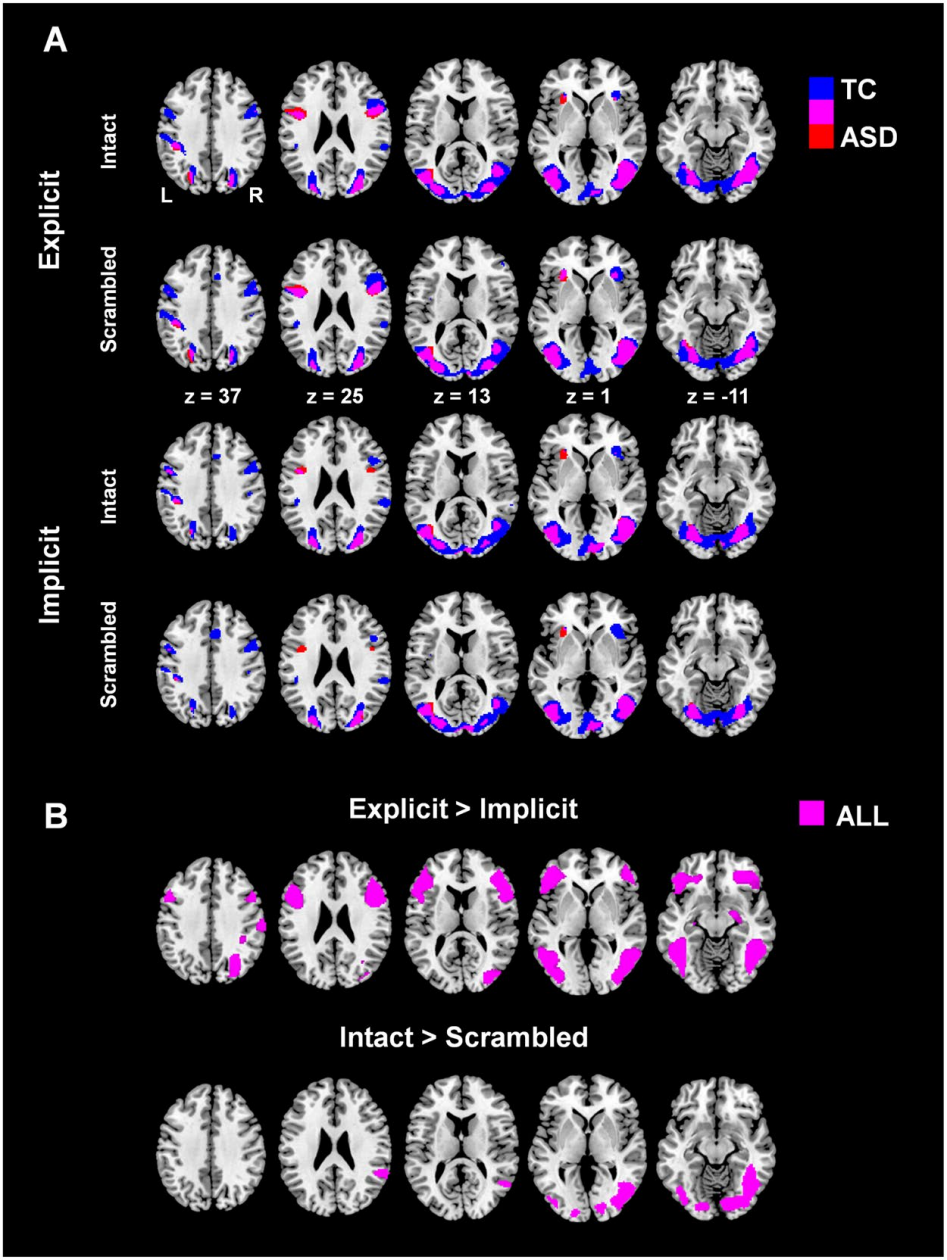
Whole-brain analysis of task-related brain activity during biological motion processing. Panel A visualizes for each group (ASD, TC) task-related brain activity (>fixation) during explicit and implicit biological motion processing, separately for intact and scrambled PLDs. Panel B visualizes across groups (ASD and TC) differences in task-related brain activity for explicit versus implicit processing (upper panel) and for processing of intact versus scrambled PLD (lower panel). (whole-brain p < 0.05 voxel-wise threshold, FWE-corrected).

Figure 2A visualizes active brain regions during explicit and implicit biological motion processing ( >fixation), separately for intact and scrambled PLDs. In all conditions, both TC (blue) and ASD groups (red) activated a bilateral fronto-parietal network corresponding to the action observation or mirror network (including regions in the inferior frontal gyrus (IFG) pars triangularis and opercularis; premotor cortex in precentral gyrus (Brodmann area (BA) 6); supplementary motor area (SMA); and postcentral gyrus (inferior parietal cortex)). Also several visual areas were identified in occipital cortex (inferior occipital gyrus (IOG); calcarine gyrus) and temporal cortex (inferior temporal gyrus; superior temporal gyrus; middle temporal gyrus; fusiform gyrus) (whole-brain p < 0.05 voxel-wise threshold, FWE-corrected).

Across groups, explicit processing of biological motion elicited higher activation compared to implicit processing of the fronto-parietal network and several occipito-temporal visual areas (whole-brain p < 0.05 voxel-wise threshold, FWE-corrected) (Fig. 2B, upper panel). Further, across groups, intact versus scrambled PLDs elicited higher activations mostly in visual areas in the occipital lobe (IOG) and temporal lobe (fusiform gyrus and right superior temporal gyrus (STG) (whole-brain p < 0.05 voxel-wise threshold, FWE-corrected) (Fig. 2B, lower panel).

### Group differences in task-related activity during biological motion processing

#### Whole-brain analysis

As seen in Fig. 2A, activation clusters were generally larger in the TC group compared to the ASD. However, direct comparison of groups at the whole-brain level failed to reveal significant differences in regional activation after correction for multiple comparisons at the cluster level (whole-brain p < 0.001 voxel-wise threshold; p < 0.05 cluster-wise threshold, FWE-corrected).

#### Regional analysis in bilateral STS

**Figure 3:**
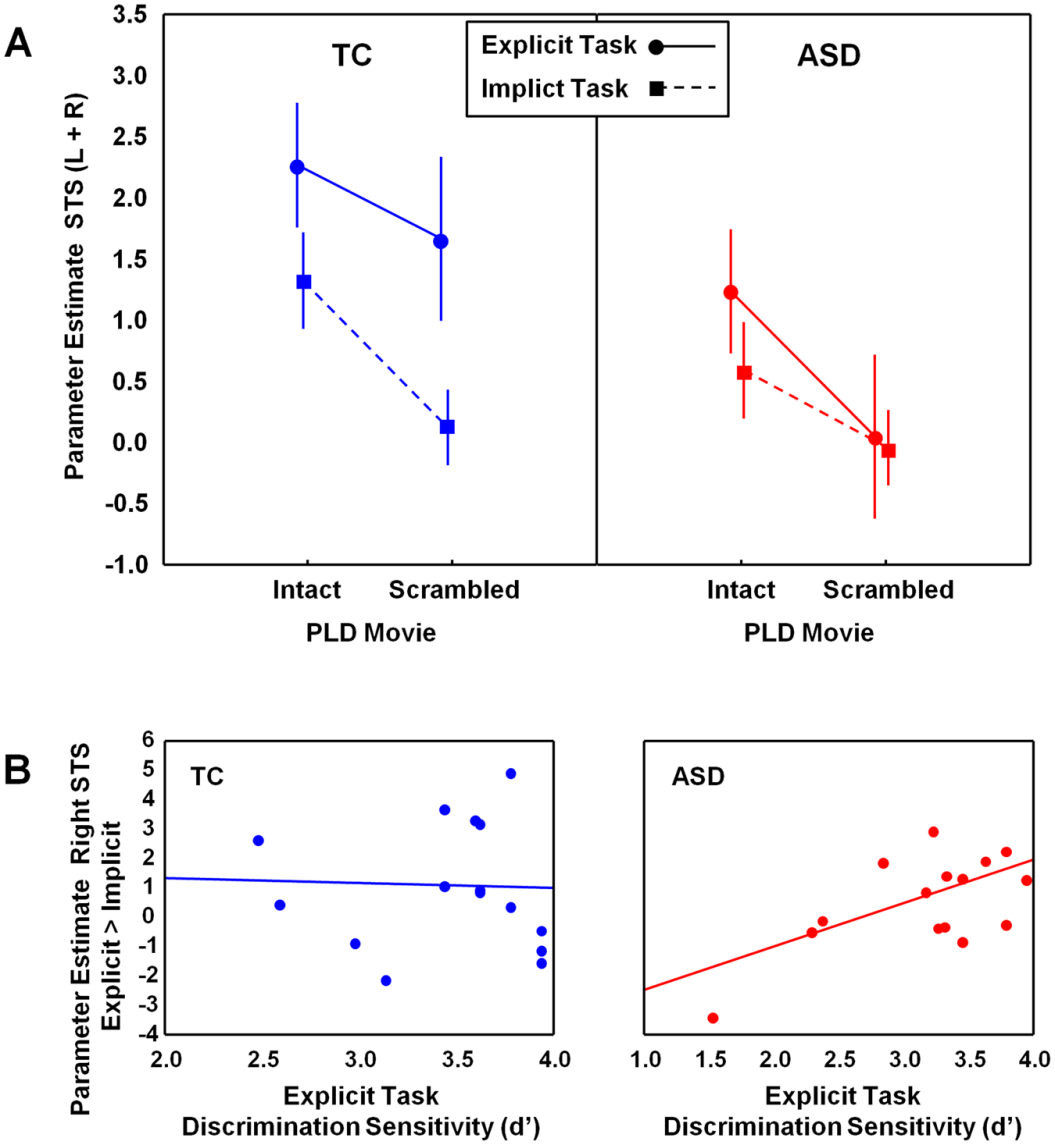
Regional analysis of task-related brain activity during biological motion processing in bilateral STS. Panel A visualizes for each group (ASD, TC) parameter estimates of task-related brain activity (>fixation) in bilateral STS during explicit and implicit biological motion processing, separately for intact and scrambled PLDs. Panel B visualizes for each group (ASD, TC) the relationship between discrimination sensitivity (d’) and right STS parameter estimates of task-related brain activity for explicit biological motion processing (>implicit). Vertical bars denote +/− standard errors.

Region-of-interest (ROI) analyses were performed within bilateral STS to specifically explore differential activations for each factor level and group using a mixed-effects model with the factors ‘group’ (TC, ASD), ‘ROI’ (right STS, left STS), ‘task condition’ (explicit, implicit) and ‘PLD movie’ (intact, scrambled). Figure 3A displays the activation patterns separately for each group (across left and right ROIs). Supplementary Figure 2 visualizes the activation patterns separately for each ROI.

First, a **main effect of ‘ROI’** (F(1, 224) = 15.12; p < 0.001) indicated that activity was generally higher in right STS compared to left STS (across stimulus type and task conditions) which is in accordance with previous studies adopting similar PLD stimuli^21, 26, 27^ (Supplementary Figure 2).

In terms of group differences, a significant **main effect of ‘group’** was revealed (F(1, 28) = 10.65; p < 0.01), indicating that overall, the TC group displayed higher activations, compared to the ASD group, both in left and right STS and across task conditions and stimuli (intact, scrambled), (Fig. 3A). With respect to the type of presented stimuli, a significant **main effect of ‘PLD movie’** (F(1, 196) = 23.17; p < 0.001) indicated that STS activity was generally higher for viewing intact, compared to scrambled PLD motion and this effect was not significantly modulated by group (‘group × movie type’; F(1, 196) = 0.004; p = 0.95). This indicates that although activity was generally lower in the ASD group, individuals with ASD were able to show a significant differential recruitment of the STS regions in response to intact versus scrambled PLD motion (similar to the TC group).

With respect to **task condition**, a significant main effect was revealed (F(1, 196) = 16.16; p < 0.001), indicating that STS activity was higher for explicit, compared to implicit processing. Here however, a significant ‘group x task condition’ interaction (F(1, 196) = 6.203; p = 0.01) was additionally revealed, indicating that only in the TC group, explicit processing of the presented PLD yielded a significant augmentation of activity in the STS regions (compared to implicit processing) (p < 0.001), whereas in the ASD group, activity levels were not differentially modulated for implicit versus explicit processing of the PLD stimuli (p = 0.31) (Fig. 3A).

Note that a similar main effect of ‘group’ (F(1, 27) = 8.72; p < 0.01) and ‘group × task condition’ interaction effect (F(1, 188) = 4.28; p = 0.04) was revealed when the primary statistical analyses were performed without the TC participant with a high self-reported SRS-score, indicating that inclusion/exclusion of this participant did not qualitatively alter the pattern of results (see Supplementary Figure 1C).

Brain-behavior correlation analyses were performed to directly explore whether the ability to differentially modulate STS activity upon *explicit versus implicit* processing was related to variations in performance on the biological motion discrimination task. Only in the ASD group (r = 0.59; p = 0.02), not in the TC group (r = −.04; p = 0.90), a positive relationship was identified between differential activity of the right STS (explicit > implicit) for viewing the *intact* PLD stimulus and discrimination sensitivity (d’), indicating that patients with limited task-specific augmentation of right STS activity (‘intact’ explicit > implicit) showed a reduced ability to discriminate biological motion (Fig. 3B). Note however that one case of the ASD group exhibited a strong influence on this relationship (Cook’s distance = 1.35), and that secondary analyses without this case failed to replicate the significant positive relationship (r = 0.31; p = 0.27).

No significant relationships were revealed between recruitment of the right STS for viewing the *scrambled* PLD and behavioral performance (d’) or between recruitment of *left* STS (intact or scrambled) and behavioral performance (d’).

### Effective connectivity of right STS during biological motion processing

**Figure 4:**
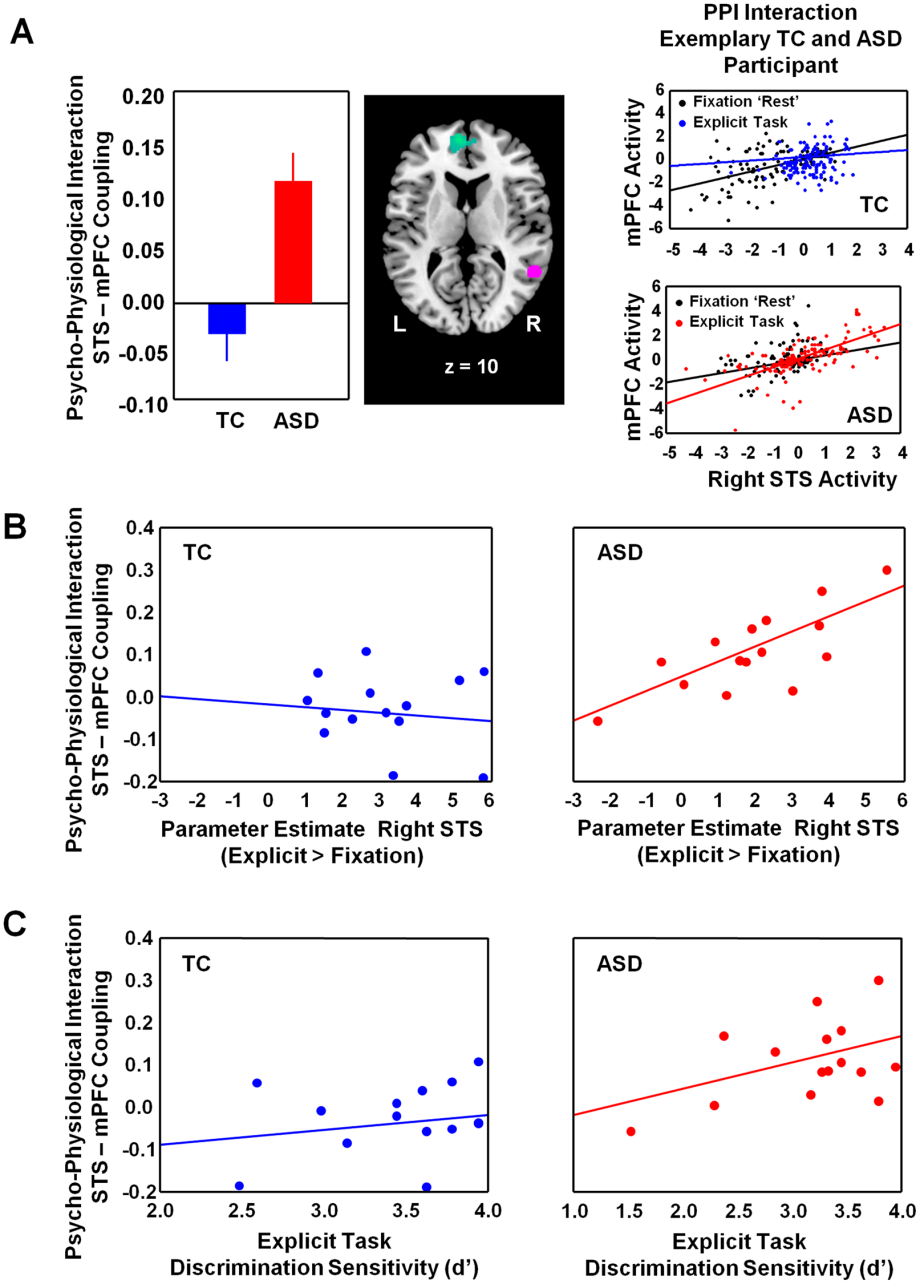
Psycho-physiological interaction analysis. Panel A visualizes the group difference in effective connectivity of right STS (purple) with a cluster in medial prefrontal cortex (mPFC) (blue-green) during explicit biological motion processing (>fixation). The right panel visualizes the psycho-physiological interaction for an exemplary participant of the ASD and TC group. Panel B visualizes for each group (ASD, TC) the relationship between coupling (effective connectivity) of right STS with mPFC and parameter estimates of task-related brain activity in right STS (explicit > fixation). Panel C visualizes for each group (ASD, TC) the relationship between coupling (effective connectivity) of right STS with mPFC and discrimination sensitivity (d’).

A psycho-physiological interaction (PPI) analysis was conducted to assess group differences in effective connectivity of right STS during explicit biological motion processing (versus fixation). At a whole-brain level, the ASD - as compared to the TC group - was shown to display stronger coupling between right STS and a cluster in the medial prefrontal cortex (mPFC) (MNI peak coordinates: 5, 54, 14) (Fig. 4A) (with outlier TC participant: F(1, 28) = 18.47; p < 0.001) (without outlier TC participant: (F(1, 27) = 19.63; p < 0.001). Inspection of the differential coupling pattern showed that in the TC group, right STS-mPFC coupling was relatively diminished during explicit biological motion processing (compared to fixation), whereas in the ASD group, right STS-mPFC coupling was enhanced during explicit biological motion processing (compared to fixation) (Fig. 4A, right panel, visualization of exemplary subjects).

Interestingly, only in the ASD group (r = 0.74; p < 0.01), not in the TC group (r = −0.10; p = 0.72), the extent of right STS-mPFC coupling was significantly associated with increased right STS activity during explicit biological motion processing (>fixation) (Fig. 4B). Further, in the ASD group (r = 0.43; p = 0.08), not in the TC group (r = 0.16; p = 0.60), stronger right-STS-mPFC coupling was tentatively associated with higher discrimination sensitivity (greater ability to discriminate intact from scrambled PLDs) (Fig. 4C).

### Relationship with self-reported SRS-scores

Correlation analyses with self-reported SRS-scores are reported in Supplementary Figure 3. In the TC group, not in the ASD group, tentative relationships were revealed between self-reported SRS-scores and (i) the extent of right STS-mPFC effective coupling (r = 0.54; p = 0.06); as well as discrimination sensitivity on the explicit task (r = −0.49; p = 0.089) (Supplementary Figure 3). Note however that both relationships were predominantly driven by the TC participant with a high SRS score (without outlier TC participant, both p > 0.2).

## Discussion

In the present study, the involvement of the superior temporal sulcus (STS) in explicit and implicit biological motion processing was investigated in ASD and TC participants. Effective connectivity of right STS during biological motion processing was also explored.

Across groups, we identified the recruitment of bilateral fronto-parietal areas and temporo-occipital areas both during explicit and implicit PLD biological motion processing. Although the recruited network for biological motion processing was overall similar across groups, region-wise analysis showed that patients with ASD displayed significantly lower activations in bilateral STS regions compared to the TC group, especially during explicit biological motion processing requiring the active discrimination of intact from scrambled PLD.

In terms of stimulus type, both ASD and TC participants showed higher STS activity for processing intact versus scrambled PLD. However, in terms of task condition, only in the TC group, explicit processing of the presented PLD yielded a significant augmentation of activity in the STS regions (compared to implicit processing). In the ASD group, on the other hand, activity levels were similar for implicit versus explicit processing of the PLD stimuli. Furthermore, in the ASD group, inter-individual variance in the extent of differential right STS recruitment (explicit > implicit processing) was shown to be related to differences in discrimination sensitivity, indicating that patients with low (task-dependent) STS recruitment showed a reduced ability to discriminate intact from scrambled PLD (although note that this relationship was mainly driven by one outlier subject).

Together, these results provide indications that while the basic (implicit) neural processing of biological motion might be intact, patients with ASD may show a reduced ability to effectively recruit the adequate neural loci when explicitly prompted to draw inferences from the biological motion stimuli. Overall, our findings provide support to the ‘social motivation theory’ of ASD^50^, postulating that patients with ASD may be primarily characterized by a reduced ability to recruit the relevant socio-cognitive skills to perform social tasks (due to impairments in social attention orienting), but that the underlying socio-cognitive resources may be relatively spared. Particularly, as theorized by Chevalier *et al*., (2012), the ‘social motivation theory of ASD’ posits that impairments in orienting attention to social stimuli (rather than social processing per se) may constitute the primary cause of ASD-related disruptions in socio-communicative skills, namely by depriving patients with ASD of adequate social learning experiences during development.

The suggested relevance of impairments in social attention orienting in ASD is further supported by results from our psycho-physiological interaction (PPI) analysis exploring changes in effective connectivity of the STS during biological motion processing. This analysis identified a cluster in medial prefrontal cortex (mPFC) - a key region in ‘top-down’ attention regulation - to be differentially coupled to right STS in ASD and TC individuals. More specifically, in the TC group, a relative de-coupling was observed during the explicit biological motion task, whereas, in the ASD group, an increase in coupling between right STS and mPFC was evident during active biological motion processing. As discussed in the introduction, the STS is hypothesized to form an integral part of distinct social brain networks subserving action/emotion understanding (action observation network or mirror neuron system) and theory of mind or mentalizing processes (amygdala-prefrontal network)^28^. While our adopted biological motion processing paradigm predominantly recruited regions of the action observation network (including inferior parietal and inferior frontal/premotor cortices, along with occipital regions and temporal regions such as STS), results from the psycho-physiological interaction analysis showed a heightened coupling between STS and a core region of the mentalizing network (namely mPFC) in the ASD group. Prior studies showed that the mentalizing network - and mPFC in particular - is robustly activated when participants engage in complex social tasks, such as observing social interactions embedded in cartoon triangles^52^, inferring other people’s intentions from stories or pictures of human actions^53–55^; or playing interactive games that requires consideration of the opponents’ beliefs^56^. In this context and by virtue of its connections to the limbic system (e.g. amygdala), the mPFC has been hypothesized to form a key region of a cognitive ‘top-down’ attentional control mechanism, important for regulating attention and behavioural responses towards self-relevant events in the surrounding environment^57^. Although indirectly, the observation of increased coupling between right STS and a core mentalizing region during biological motion processing in the ASD group may reflect a compensatory mechanism for facilitating attention orienting during explicit task requirements (i.e., resulting in an increased ability to discriminate biological from non-biological motion stimuli in patients with increased STS-mPFC coupling). Indeed, in the ASD group, the extent of continued coupling was associated with heightened task-induced right STS activation which in turn was significantly associated with the ability to discriminate intact biological motion from scrambled dot motion.

Overall, our findings converge with previous studies that reported differences between individuals with ASD and typically-developing populations in terms of brain activation within the STS during *explicit* biological motion processing^7, 18, 46–49^. However, the observation that stimulus-dependent modulations of STS activity (intact versus scrambled) were comparable in the ASD group and TC group, and that group differences in STS activity were only present during explicit, but not during implicit biological motion processing, provides strong indications that prior reports of altered STS recruitment may primarily reflect an inadequacy of ASD patients to *recruit* the necessary neural loci (STS) upon task demand, rather than a generalized aberration of neural processing at the level of STS. The relative importance of attention orienting for recruiting the relevant resources upon task demand was further underlined by the observation that connectivity between STS and mPFC, a key region in ‘top-down’ attention regulation, effectively modulated the recruitment of STS during explicit task requirements in the ASD group.

## Methods

### Participants

Fifteen high-functioning adult males with an autism spectrum disorder (ASD) (aged, 21.7 ± 4.0 years (mean ± standard deviation)) and 15 typically developed controls (TC) (23.3 ± 2.9 years) participated in the present study (Table 1). Groups were matched for age, gender, full-scale intelligence quotient (IQ) and performance IQ (Table 1) (Ward 7-subtest of the Wechsler Adult Intelligence Scale-III^58, 59^). All ASD participants were recruited from the Autism Expertise Centre at the Leuven University Hospital. They previously had taken part in a larger family study conducted by the Leuven Autism Research Consortium (LAuRes)^60^ during which a multidisciplinary team (child psychiatrist and/or expert neuropediatrician, psychologist, speech/language pathologist and/or physiotherapist) formulated a DSM-IV-TR diagnosis of autistic disorder^61^. Diagnosis was obtained by combining information from unstructured direct observation, semi-structured parent interview (developmental, dimensional and diagnostic interview (3di)^62^) as well as review of prior history and parent screening questionnaires. For all ASD participants, parents completed the Dutch version of the Social Responsiveness Scale (SRS)^63^, a 65-item questionnaire developed to assess a wide range of interpersonal behavior, communication and repetitive/stereotypic behavior characteristic of ASD^63, 64^. Only participants with prior ASD diagnosis and a parental total SRS score (raw) above 60 were included. Six (of 15; 40%) participants with ASD had a total parental SRS T-score within the mild to moderate range (60 through 75) indicating clinically significant impairment associated with mild to moderate interference in everyday social interactions. Nine (of 15; 60%) participants with ASD had total SRS T-scores within the severe range (76 or higher), indicative of severe interference in everyday social interactions. Total parent SRS scores (raw and T-scores) are listed in Table 1. For all participants (ASD and TC), self-reported SRS scores (adult-Dutch version^63^) were assessed. Overall, self-reported SRS scores were significantly higher in the ASD, compared to the TC group (Table 1 and Supplementary Figure 1A).

**Table 1:** Characteristics of the groups.

Gender	ASD (n = 15)		TC (n = 15)		t-value	p
	Mean	SD	Mean	SD		
	All males		All Males			
Age in years	21.7	4.0	23.3	2.9	1.2	0.22
Verbal IQ	109.1	12.9	117.4	9.9	2.0	0.06
Performance IQ	105.6	19.3	109.1	17.7	0.5	0.61
Full Scale IQ	107.9	13.9	114.8	12.8	1.4	0.16
Total SRS - parental report (raw)	91.5	28.5				
Total SRS - parental report (T)	77.0	12.1				
Total SRS - self report (raw)	76.5	24.2	43.6	21.7	−4.3	<0.001
Social Awareness	9.3	3.0	6.9	2.9	−2.3	<0.05
Social Cognition	14.7	4.7	7.3	4.9	−4.3	<0.001
Social Communication	25.0	6.7	14.4	8.2	−3.9	<0.001
Social Motivation	14.7	4.9	7.9	4.8	−3.8	<0.001
Autistic Mannerisms	12.9	4.9	7.1	4.5	−3.3	<0.05
Head Motion (mean FD)	0.20	0.08	0.19	0.10	−0.31	0.76

Note however that within the TC group, one participant was identified as an outlier, with a high self-reported SRS-score of 103 (see Supplementary Figure 1). For completeness and to verify the impact of this participant on the obtained pattern of results, all primary statistical analyses are reported with and without exclusion of this participant.

All methods were carried out in accordance with relevant guidelines and regulations and the study protocol was approved by the local Ethical Board (UZ KU Leuven – Research). Informed consent was obtained from all participants according to the Declaration of Helsinki. None of the participants took psychoactive medications at the time of the scan.

### fMRI Data Acquisition

Anatomical and task-related fMRI images were acquired on a 3.0 Tesla Philips MR scanner (Best, The Netherlands) with an 8-channel phased-array head coil. Scan sessions started with the acquisition of the anatomical scan, followed by the two task-related fMRI runs. Resting-state fMRI scans and other task-based fMRI images were also acquired from the same subjects which are reported in ref. 7.

Anatomical imaging consisted of a high resolution structural volume acquired using a coronal three-dimensional turbo field echo T1-weighted sequence with the following parameters: 182 contiguous coronal slices covering the whole brain and brainstem, slice thickness = 1.2 mm; repetition time (TR) = 9.7 ms; echo time (TE) = 4.6 ms; matrix size = 256 × 256; field-of-view (FOV) = 250 × 250 mm²; in-plane pixel size = 0.98 × 0.98 mm²; acquisition time = 6 min 38 s.

For the two task-related fMRI scans a T2* weighted gradient echo - echo planar imaging (GE-EPI) sequence was used with the following parameters: TR = 3000 ms; TE = 33 ms; matrix size = 80 × 80; FOV = 230 mm; flip angle 90°; slice thickness 4 mm, no gap; axial slices = 35; 151 functional volumes (148 + 3 first functional volumes discarded for equilibrium of longitudinal magnetization); total acquisition time = 7 min 33 sec.

### Task-based fMRI paradigm

During task-based fMRI, participants were presented with **point light displays** (PLDs) of biological motion which were either ‘intact’ or ‘scrambled’. The adopted PLDs were based on motion capture data as described in previous work from our group^6, 65^. In short, twelve reflective markers attached to the joints of the ankles, the knees, the hips, the wrists, the elbows, and the shoulders of a male and female actor were tracked using an eight-camera VICON system (capturing system measuring at 100 Hz, Oxford Metrics, Oxford, UK) while the actors performed three actions (walking, jumping, kicking) in four different ‘emotional states’ (neutral, happy, sad, angry). In the recorded PLD movie files (duration 3 sec), marker positions were visible as twelve moving white spheres on a black background (Fig. 1A); presented from three different viewing perspectives (front view, 90° side view and 45° intermediate view). For the current fMRI task paradigm, a subset of 40 PLD stimuli were selected that were shown to be reliably identified as ‘biological’, but below ‘ceiling’ performance in a normative sample of thirty-seven control participants (15 males/22 females) (described in ref. 65). In the included set of ‘biological’ PLD stimuli, 50% of the movies showed the male model; while the other 50% showed the female model. The emotional state of the PLD model was either ‘neutral’ (25% of the movies), ‘happy’ (25%), ‘sad’ (25%) or ‘angry’ (25%). The ‘action’ of the PLD stimulus was either ‘walking’ (40% of the movies); ‘kicking’ (30%) or ‘jumping’ (30%); presented from a ‘front view’ (30% of the movies); a ‘side view’ (32.25%) or an ‘intermediate view’ perspective (32.25%). For each of the 40 intact ‘biological’ PLD movies, a ‘scrambled’ version was created which consisted of the same individual dots, undergoing the same local trajectories as in the intact PLD, however with the initial starting position of the 12 individual dots randomly permutated to a different starting position (Fig. 1A).

During fMRI scanning, the set of 80 PLD movies (40 intact, 40 scrambled) was randomly presented to the participants in an ‘explicit’ and ‘implicit’ task condition (i.e., total of 160 presented PLD movies). Particularly, participants completed two task-based fMRI runs, each consisting of eight task blocks of 10 trials (2 runs × 8 blocks × 10 movies). In half of the blocks (8) attention was focused explicitly towards the biological motion content conveyed by the stimuli, by instructing the participants to indicate as fast and accurate as possible whether the presented PLD represented ‘a person’ or ‘not a person’ (‘explicit’ task condition). In the other half of the blocks (8), participants were instructed to indicate color changes in the moving PLD, such that in this task condition, attention was not explicitly focused towards the biological content conveyed by the PLD movies (‘implicit’ task condition). In all PLD movies, one out of the twelve dots briefly (0.5 s) changed color to ‘red’ or ‘green’ and participants had to indicate the color change (note that the dot that changed color was random across trials).

All trials lasted 4 s, such that stimulus presentation was jittered with respect to image acquisition (TR = 3 s). Each task-based run consisted of 8 blocks (40 sec/block) separated by 16 s fixation blocks, during which participants fixated on a white cross. At the end of each run, a final fixation block was presented for 12 sec. As such, the total duration of each task-based run was 444 sec (8 × task blocks (40 sec) + 7 × fixation blocks(16 sec)) + 1 final fixation block (12 sec), covered by 148 functional volumes (TR = 3 sec). Task instructions were provided verbally and on the monitor at the start of each task block. Response options were displayed at the bottom of the screen, which corresponded to response buttons of a response box. Participants were instructed to respond as fast and accurately as possible and to use the right index and middle finger for button pressing. Prior to the scanning session, subjects practiced the task conditions (explicit and implicit) during a training run (7 min 33 sec) inside a mock scanner.

### Data analysis: Behavioral Performance

Correct reaction times (RTs) and response accuracy were assessed using E-Prime-software (Psychological Software Tools). For each task condition (explicit, implicit), a performance index was calculated (Accuracy/RT).

For the explicit biological motion task, we also explored the discrimination sensitivity (d-prime or d’) and response bias (criterion or c). In signal detection theory, discrimination sensitivity is conceived as detecting a ‘signal’ compared to another (noise) signal; i.e., in our task, detecting ‘biological’ PLD from ‘scrambled’ PLD. The hit rate (H) was calculated as the proportion of ‘biological’ trials to which the subject responded ‘biological’ (P(biological, biological)). The false alarm rate (F) was calculated as the proportion of ‘scrambled’ trials to which the subject responded ‘biological’ (P(biological, scrambled)). In general, a subject’s sensitivity is higher if the difference between H and F is larger (e.g., according to the pair (H,F), the perfect subject’s performance is (1,0), while a random subject has H = F and a subject who always answered ‘biological’ has (1,1)). The statistic d’ represents this distance by calculating the difference between the z-transforms of the hit rate (H) and false alarm rate (F) [d’ = z(H) −  z(F)]. A higher d’ indicates that the signal can be more readily detected.

Note that for the same d-prime score, collectively higher or lower levels of z(H) and z(F) will reflect differences in a subject’s criterion level or response bias. To evaluate the common level of z(H) and z(F), we additionally calculated the statistic ‘criterion’ as [c = −1/2[z(h) + z(f)]]. Based on this measure, a ‘strict’ criterion or bias to indicate the stimulus as ‘non-biological’ would be reflected by overall low levels of z(H) and z(F) (criterion higher than zero); a ‘low’ criterion or bias to respond ‘biological’ would be reflected by overall high levels of z(H) and z(F) (criterion smaller than zero); and finally a mid-way criterion or unbiased response would be reflected by c = 0.

Repeated-measures Analysis of Variance (ANOVA) on the performance index was conducted with the between-subject factor ‘group’ (ASD, TC) and the within-subject factor ‘task condition’ (explicit, implicit) to explore behavioral group differences in detecting biological motion (explicit biological motion task) or detecting color changes (implicit condition). Due to violations of the normality assumption (Kolmogorov–Smirnov tests), non-parametric Mann-Whitney U Tests for independent samples were used to explore group differences (ASD, TC) in discrimination sensitivity (d’) on the explicit biological motion task. A parametric T-test for independent samples was used to assess group differences in response bias (criterion). Statistics were performed using Statistica 10 (StatSoft. Inc. Tulsa, USA). The significance level was set at p < 0.05 for all analyses.

### Data analysis: Task-based fMRI

SPM 8 was used for image preprocessing and statistical analyses (Wellcome Department of Cognitive Neurology, London, UK).

Task-related functional images were spatially realigned and unwarped, corrected for differences in slice acquisition time by temporal interpolation to the middle slice (reference = 17), normalised to the standard EPI template of the Montreal Neurological Institute (MNI), resampled into 2 mm isotopic voxels and spatially smoothed with an isotropic 8 mm full-width-at-half-maximum Gaussian kernel. A high-pass filter with a cutoff of 184 s was used to remove slow signal drifts.

Head motion, assessed as mean frame-wise displacement (FD) was minimal and was not significantly different between groups (t(28) = −0.31; p = 0.76) (Table 1).

For each subject and run, a general linear model^66^ was calculated including 4 epoch regressors, representing the 2 × 2 levels of the factors ‘task condition’ (explicit, implicit) and ‘PLD movie’ (intact, scrambled). We also included the time-courses of the rest blocks (fixation) as an implicit regressor and the time series of the six realignment parameters as regressors of no interest.

For each subject, contrast images were calculated for each factor level (>fixation) which were subjected to second-level random-effect models to perform group level analyses. For each group (TC, ASD), one-sample t-tests were implemented to identify regions with reliable activity for each factor level [‘task condition’ (explicit, implicit) x ‘PLD movie’ (intact, scrambled)] using a whole-brain voxel-wise threshold of p < 0.05 family wise error (FWE) corrected for multiple comparisons. To explore group differences at the whole-brain level, two-sample t-tests were implemented using a whole-brain p < 0.001 voxel-wise threshold and a cluster-wise threshold of p < 0.05, FWE-corrected. The two-sample t-test analyses were masked with a factor-specific conjunction mask in order to include only regions that are active to the specific factor in both groups (conjunction map, thresholded at p < 0.05).

Considering abundant reports on the involvement of the (posterior) superior temporal sulcus (STS) in biological motion processing (specifically right STS), region-of-interest (ROI) analyses were performed to explore differential activations within bilateral STS for each factor level and group. To obtain an unbiased identification of ROI coordinates, a term-based meta-analysis was performed in *Neurosynth* (http://neurosynth.org/) generating a meta-analytic brain map of regions relevant to the term ‘biological’ (221 studies - reverse inference p < 0.01 FDR corrected). As visualized in Supplementary Figure 4, the Neurosynth-based meta-analytic brain map only identified two regions in bilateral STS, which underlines the specific involvement of these regions (particularly right STS) in biological processing (note that the neurosynth-identified cluster in right STS was comparably larger compared to the left STS cluster). Two 10 mm radius spherical ROIs were centered on the peak coordinates of the right (MNI: 55, −52, 10) and left STS cluster (MNI: −55, −52, 12) and the average contrast estimate within each ROI was extracted for each subject to perform region-wise group-level mixed-effects analyses with the factor ‘group’ (TC, ASD) modeled as random effect and the factors ‘ROI’ (right STS, left STS), ‘task condition’ (explicit, implicit) and ‘PLD movie’ (intact, scrambled) modeled as fixed effects (covariance structure; compound symmetry).

As reported in more detail in the result section, the regional analysis of bilateral STS showed a main effect of ‘ROI’ (region-of-interest), indicating that activity was generally higher in right STS compared to left STS (across stimulus type and task conditions), a finding that accords with several prior studies reporting a relative right STS specialization for biological motion processing^21, 26, 27^.

To further explore the involvement of right STS in biological motion processing, a psycho-physiological interaction (PPI) analysis was conducted to investigate changes in coupling (effective connectivity) between activity in the right STS (spherical seed, MNI coordinates: 55, −52, 10) and any other voxel in the brain for performing the explicit biological motion task, compared to rest (fixation). A two-sample t-test was implemented to explore group differences in effective connectivity using a whole-brain voxel-wise threshold of p < 0.001 and a cluster-wise threshold of p < 0.05 (FWE-corrected).

## Electronic supplementary material

Supplementary Information
