# Supplementary material for: Neural processing of biological motion in autism: An investigation of brain activity and effective connectivity
Source: Sci Rep. 2017 Jul 17;7:5612. doi: 10.1038/s41598-017-05786-z (PMC5514051; doi:10.1038/s41598-017-05786-z)

Supplementary Information

Neural processing of biological motion in autism: An investigation of brain activity and effective connectivity

Kaat Alaerts, Stephan Swinnen, Nicole Wenderoth

Supplementary Figures

- **Supplementary Figure 1:** Group differences in self-reported SRS-scores and analysis of behavioral and neural group effects without TC participant with a high self-reported SRS-score
- **Supplementary Figure 2**: Regional analysis of task-related brain activity during biological motion processing in left and right STS
- **Supplementary Figure 3:** Relationship with self-reported SRS-scores
- **Supplementary Figure 3:** Region-of-interest (ROI) definition for left and right superior temporal sulcus (STS).

Supplementary Figure 1

Group differences in self-reported SRS-scores and analysis of behavioral and neural group effects without TC participant with a high self-reported SRS-score

Panel A visualizes the group difference in self-reported SRS-scores (Social Responsiveness Scale) between the ASD and TC groups. Note that within the TC group, one participant was identified as an outlier, with a high self-reported SRS-score of 103 (i.e., score larger than Q3 ± 1.5 (Q3-Q1), with Q1 and Q3 being the first and third quartile (Statistica 10; StatSoft. Inc. Tulsa, USA)).

Panel B visualizes the group-related differences in behavioral performance without the outlier TC participant.

Panel C visualizes the group-related differences in STS task-related activity during biological motion processing without the outlier TC participant.

Overall, exclusion of this participant did not qualitatively alter the reported pattern of results (as visualized in Figure 1B and 3A).

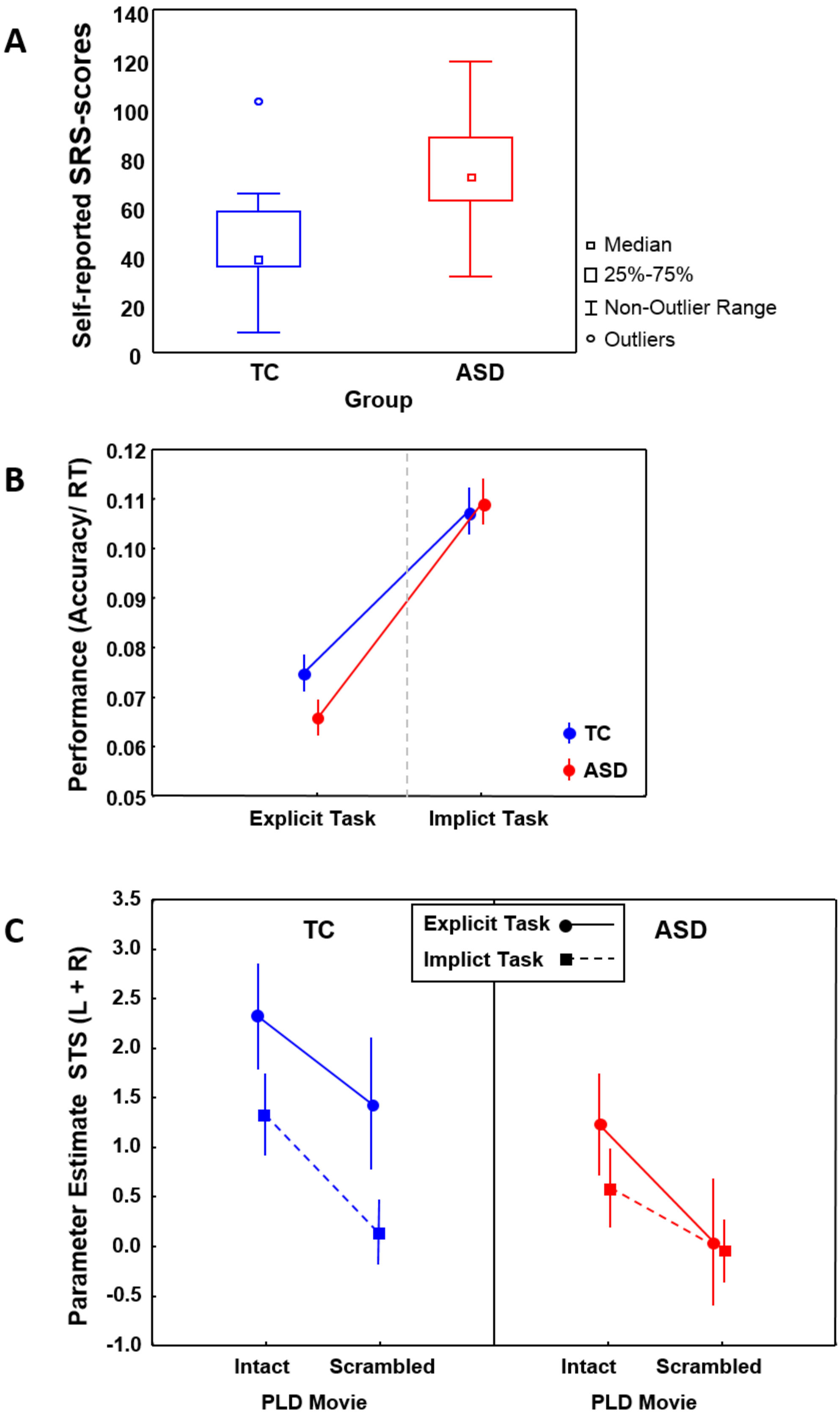
A
Self-reported SRS-scores
140
120
100
80
60
40
20
0
TC
ASD
Group
Median
25%-75%
Non-Outlier Range
Outliers
B
Performance (Accuracy/ RT)
0.12
0.11
0.10
0.09
0.08
0.07
0.06
0.05
Explicit Task
Implict Task
TC
ASD
C
Parameter Estimate STS (L + R)
3.5
3.0
2.5
2.0
1.5
1.0
0.5
0.0
-0.5
-1.0
TC
ASD
Explicit Task
Implict Task
Intact
Scrambled
PLD Movie
Intact
Scrambled
PLD Movie

Supplementary Figure 2

Regional analysis of task-related brain activity during biological motion processing in left and right STS

Visualization of parameter estimates of task-related brain activity in left and right STS (> fixation) for each group (ASD, TC) during explicit and implicit biological motion processing, separately for intact and scrambled PLDs.

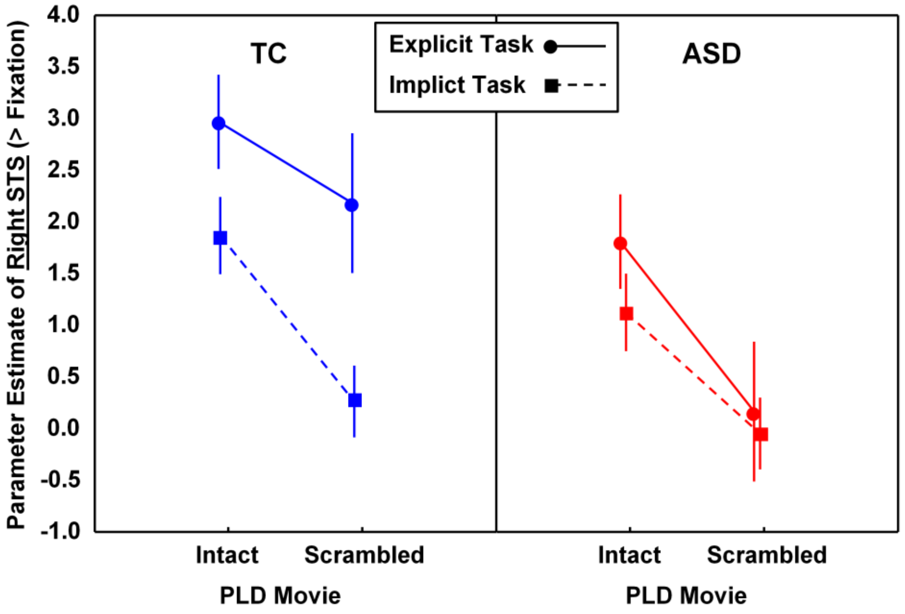

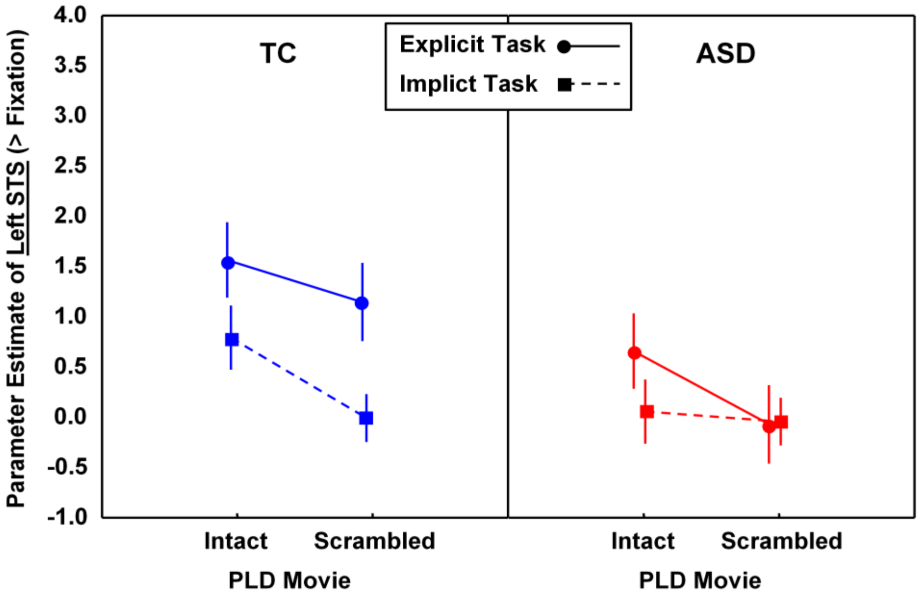

Supplementary Figure 3

Relationship with self-reported SRS-scores

Correlation analysis were performed to further explore whether a relationship exists between reports of social impairment as assessed with the social responsiveness scale (SRS-Total scores) and (i) behavioral performance (discrimination sensitivity on the explicit task (d')); (ii) STS recruitment (differential activity of the right STS for explicit > implicit processing); and (iii) effective connectivity (extent of right STS-mPFC effective coupling).

For the ASD group, no significant relationships were revealed between self- or parent-reported SRS-scores and any of these indices (all, p> .15).

In the TC group, tentative relationships were revealed between self-reported SRS-scores and (i) the extent of right STS-mPFC effective coupling (r= .54; p=.06) (**left panel**); as well as discrimination sensitivity on the explicit task (r= -.49; p=.089) (**right panel**). Note however that both relationships were predominantly driven by the TC participant with a high self-reported SRS-score (without outlier TC participant, both p > .2).

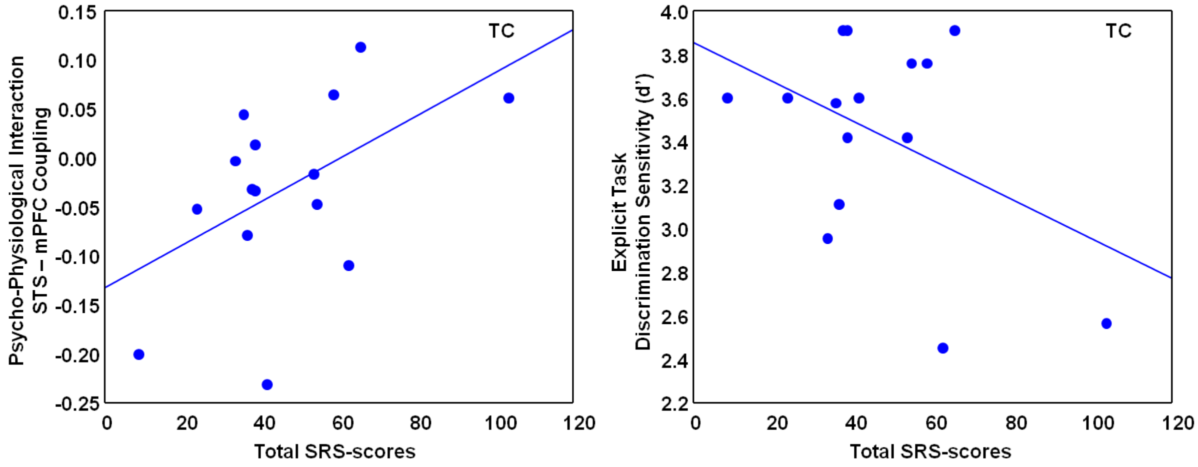

Supplementary Figure 4

Region-of-interest (ROI) definition for left and right superior temporal sulcus (STS).

To obtain an unbiased identification of ROI coordinates, a term-based meta-analysis was performed in Neurosynth (http://neurosynth.org/) generating a meta-analytic brain map of regions relevant to the term ‘biological’ (221 studies - reverse inference $p < .01$ FDR corrected), identifying two main clusters in left and right STS (yellow clusters). Two 10 mm radius spherical ROIs were centered on the peak coordinates of the right (MNI: 55, -52, 10) and left STS cluster (MNI: -55, -52, 12) (visualized in purple).

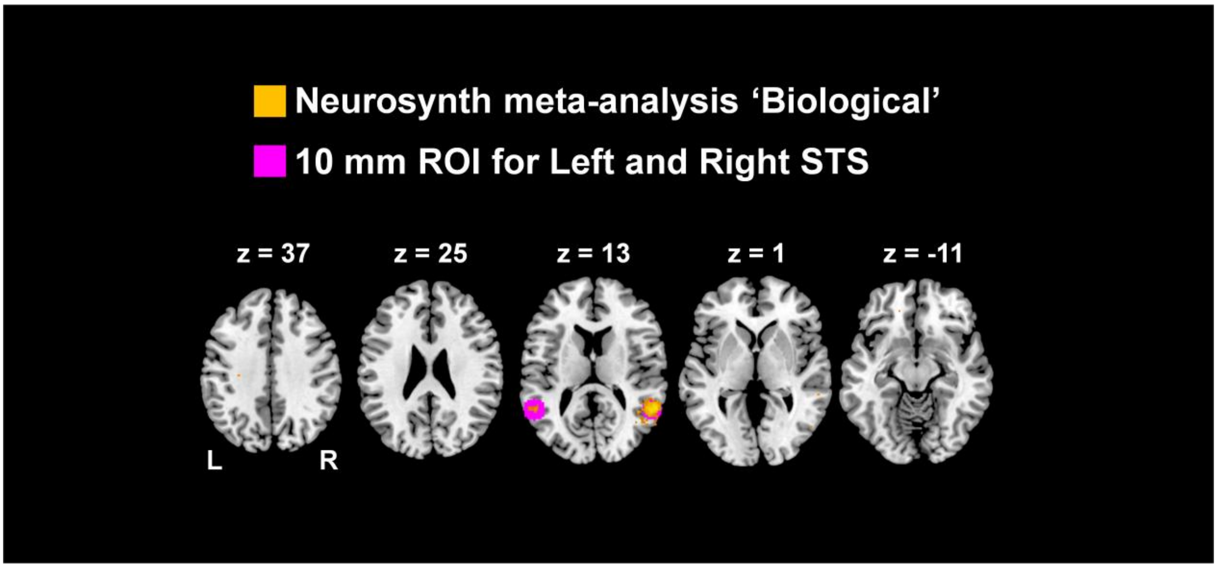

Supplement: Supplementary file 1 — Supplementary Information [file 41598_2017_5786_MOESM1_ESM.pdf]
